# Severe Osteoporosis in Larsen Syndrome—A Case Report of Bone Morphology and A Novel Filamin B (*FLNB*) Variant

**DOI:** 10.1007/s00223-025-01436-z

**Published:** 2025-10-08

**Authors:** Trine Maxel Juul, Lisbeth Koch Thomsen, Christina Møller Andreasen, Charlotte Ejersted, Lars Folkestad, Klaus Brusgaard, Stinus Hansen, Jesper Skovhus Thomsen, Thomas Levin Andersen, Anja Lisbeth Frederiksen

**Affiliations:** 1 Department of Clinical Genetics, Odense University Hospital (OUH), Odense, Denmark; 2 Molecular Bone Histology Lab, Pathology Research Unit, Department of Clinical Research, University of Southern Denmark (SDU), Odense, Denmark; 3 Department of Pathology, OUH, Odense, Denmark; 4 Department of Endocrinology M, OUH, Odense, Denmark; 5 Department of Clinical Research, University of Southern Denmark, Odense, Denmark; 6 Department of Regional Health Research, SDU, Odense, Denmark; 7 Department of Clinical Genetics, Lillebaelt Hospital, Vejle, Denmark; 8 Department of Medicine, OUH-Svendborg, Svendborg, Denmark; 9 Department of Biomedicine, Aarhus University, Aarhus, Denmark; 10 Department of Forensic Medicine, Aarhus University, Aarhus, Denmark; 11 Department of Clinical Genetics, Aalborg University Hospital, Aalborg, Denmark; 12 Department of Clinical Medicine, Aalborg University, Aalborg, Denmark

**Keywords:** Larsen Syndrome, Filamin B, FLNB, Osteoporosis

## Abstract

Larsen syndrome is a rare genetic condition characterized by facial dysmorphism and skeletal deformities. It is caused by heterozygous pathogenic variants in the Filamin B encoding gene (*FLNB*). FLNB is a cytoskeletal protein that plays a key role in bone morphogenesis; however, the skeletal phenotype of Larsen syndrome has not been described in detail. Here, we studied the skeletal presentation in two subjects with Larsen syndrome. A case-study including a 63-year-old women and her 33-year-old daughter with Larsen syndrome, both carrying a novel *FLNB* c.688G > T, *p*.(Val230Phe) variant. The bone morphologic evaluation included, radiographs, bone mineral density assessment, and high-resolution peripheral quantitative tomography (HR-pQCT). In addition, a transiliac crest bone biopsy from the mother was evaluated by µCT, histomorphometry, and in situ examination of *FLNB* expression within physiological human bone remodeling sites of controls. Both women were diagnosed with severe osteoporosis (T-score < -5). The HR-pQCT analysis showed a low trabecular bone volume, as well as a low cortical thickness compared to a healthy cohort. Histomorphometry and µCT analysis of the iliac bone biopsy confirmed low cortical thickness, and revealed a high density of small eroded and quiescent intracortical pores. The trabecular bone remodeling was not affected, while cortical remodeling events accumulated as small eroded pores and quiescent pores with an improved infilling. The *FLNB* variant is associated with low bone mineral density reflecting severe osteoporosis and an altered trabecular and cortical bone structure, while bone turnover was less affected at the time of analysis.

## Introduction

Larsen syndrome (OMIM:150250), first described by Dr. Loren J. Larsen in 1950, is a rare genetically heterogeneous disorder characterized by short stature, skeletal abnormalities—including foot deformities, scoliosis, or cervical kyphosis, malformation of the auditory ossicles leading to hearing impairment—and congenital large-joint dislocations [1]. Furthermore, individuals with Larsen syndrome may exhibit craniofacial dysmorphism, including a prominent forehead, depressed nasal bridge, hypertelorism, and telecanthus. The syndrome follows an autosomal dominant inheritance pattern and is caused by heterozygous pathogenic variants in the gene encoding Filamin B (*FLNB*) [2, 3].

Filamin proteins are dimeric and play a crucial role in the cytoskeleton by crosslinking actin filaments and converting mechanical forces into intracellular signaling [4]. Filamin B is ubiquitously expressed across various cell types but is particularly important in bone morphogenesis. Pathogenic variants in *FLNB* are associated with skeletal disorders [4].

Pathogenic variants in *FLNB* are primarily located in the action-binding domain or the repeat region within *FLNB* [2], with a genotype–phenotype correlation. Specific heterozygous *FLNB* variants cause Larsen Syndrome, while others result in lethal forms of osteochondrodysplasias, such as atelosteogenesis types I and III, and boomerang dysplasia, which are characterized by the absence or under-ossification of limb bones and vertebrae [3]. In addition, compound heterozygous or homozygous pathogenic variants are associated with a more severe form of skeletal dysplasia, known as spondylocarpotarsal synostosis syndrome [5]. Although patients with *FLNB* variants exhibit skeletal deformities, the detail of bone morphology remain to be investigated fully. Here, we report a novel *FLNB* variant and its effect on trabecular and cortical bone architecture and remodeling.

## Methods

### Study Subjects

The study subjects included a 63-year-old woman (subject 1) and her 33-year-old daughter (subject 2), both diagnosed at Department of Clinical Genetics, Odense University Hospital, and provided signed informed consent for publication.

### Genetic Analysis

Genomic DNA was isolated from peripheral leucocytes. Whole-exome sequencing was conducted using next-generation sequencing technology (Illumina NextSeq550, NimbleGen SeqCap EZ MedExome). Alignment and variant calling were performed using the BWA.MEM (Burrows-Wheeler aligner) and GATK (Broad institute) software, respectively. Genetic variants were classified in accordance to the American College of Medical Genetics and Genomics [6].

### Blood Samples

Blood samples were analyzed for p-ionized Ca^2+^, p-phosphorus (only data from subject 1), p-alkaline phosphatase, p-25-(OH) vitamin D, and p-parathyroid hormone from subject 1 (at ages 46/47 and 63 years) and subject 2 (at age 33 years). Furthermore, p-carboxy-terminal collagen crosslinks (CTx) and procollagen I intact N-terminal propeptide (P1NP) was measured on blood from subject 1 at age 63 years.

### Radiography

Subject 1 underwent conventional radiography to obtain a projection of the thorax at age 58 years. Areal bone mineral density (aBMD) of the lumbar spine (L1–L4) and total hip region was obtained using dual-energy X-ray absorptiometry (DXA, Hologic, Waltham, MA, USA).

### High-Resolution Peripheral Quantitative Computed Tomography (HR-pQCT)

From both subjects, bone geometry, volumetric bone mineral density (vBMD), and microarchitecture were assessed at the distal radius and tibia using HR-pQCT (XtremeCT, Scanco Medical AG, Brütisellen, Switzerland) at age 63 years (subject 1) and at age 33 years (subject 2). The standard patient protocol for image acquisition and analyses was applied as described previously [7]. In Brief: regions from both the distal tibia and distal radius, each including 110 slices, were obtained using an isotropic voxel size of 82 µm, an X-ray voltage of 59.4 kV and current of 900 µA, and an integration time of 100 ms. From the bone microstructural analyses, we report the trabecular bone volume per total volume (BV/TV), trabecular number (Tb.N), trabecular thickness (Tb.Th), and trabecular separation (Tb.Sp). In addition, an extended cortical evaluation analyses was applied with measurement of cortical thickness (Ct.Th) and cortical porosity (Ct.Po) [8].

For comparison, HR-pQCT results for the case subjects were compared to data obtained in a similar manner from a cohort of healthy women (n = 262) aged 20 to 80 years, who were examined in an earlier study that assessed age-related changes in bone microarchitecture [9]. The same equipment was used for both the case subjects and healthy controls, following identical protocols for image acquisition and analyses, as described above.

### Bone Biopsies/Specimens

A transiliac crest bone biopsy was collected from study subject 1 around the time of diagnosis at age 47 years, prior to bisphosphonate treatment. The biopsy was compared histologically to controls represented by transiliac crest biopsies from five healthy, age-matched women (aged 40–56 years), collected from a completely blinded biobank at the Department of Forensic Medicine, Aarhus University. After fixation, the non-decalcified biopsies were embedded in methyl-methacrylate and sectioned for the bone histomorphometric analysis.

RNA in situ hybridization analysis was performed using decalcified paraffin-embedded bone fully blinded specimens collected from the proximal femoral of three adolescent coxa valga patients undergoing corrective surgery [10], according to the approval by the Danish National Committee on Biomedical Research Ethics (Project-ID: S–2012–0193).

### Micro-Computed Tomography (µCT)

After histomorphometry, the remaining plastic-embedded tissue block was imaged using a desktop µCT scanner (µCT35, Scanco Medical AG, Brüttisellen, Switzerland) in high-resolution mode (1000 projections/180°) with an isotropic voxel size of 6 µm, X-ray voltage of 55 kVp and current of 145 µA and an integration time of 800 ms. The image data were low-pass filtered using a Gaussian filter (σ = 1.3, support = 2) in order to reduce noise and subsequently segmented with a fixed threshold filter (480.3 mg HA/cm^3^). Trabecular and cortical bone microstructure was determined with the software supplied with the µCT system. The cortical bone was analyzed similarly after swapping bone and marrow, as previously described [11]. The microstructural parameters included trabecular bone volume fraction (BV/TV), trabecular number (Tb.N), trabecular thickness (Tb.Th), trabecular spacing (Tb.Sp), connective density and tissue mineral density (TMD) for trabecular bone. Additionally, for cortical bone, the parameters included cortical porosity (Ct.Po), mean canal diameter (Ca.Dm), and canal density (Ca.Dn). The microstructural parameters were compared to those of a reference population of Danish women for trabecular bone (n = 41, aged 19–96 years) [12] and cortical bone (n = 46, aged 19–96 years) [13].

### Bone Histomorphometry

After µCT imaging, the embedded specimens were sectioned into 7.5-µm-thick sections on a Leica SM2500 microtome (Leica, Nussloch, Germany) and stained with Masson–Goldner trichrome. The sections were scanned on a NanoZoomer XR digital slide scanner (Hamamatsu, Hamamatsu City, Japan) and the images were further processed using NDP view2 (Hamamatsu, Hamamatsu City, Japan).

Within each scan, a region of interest was outlined to delineate the cortical and trabecular bone. In the cortical region, cortical thickness (Ct.Th), cortical porosity (Ct.Po), pore density (Po.Dn), and pore diameter (Po.Dm) were determined. Subsequently, all cortical pores within each cortices were classified according to their remodeling type and stage, as previously described in detail [14]. In short, the intracortical pores remodeling type was classified as either a remodeling event forming a new canal (type 1) or a remodeling of an existing canal (type 2). Additionally, the remodeling stage of the pores was characterized as either quiescent (Q pores) or non-quiescent (non-Q pores) with the latter representing pores with either eroded (E pores), eroded-formative (EF pores), or formative (F pores) surfaces. In the quiescent pores/osteons, which reflect a terminated remodeling cycle, the osteon diameter (O.Dm) and pore diameter (Po.Dm) were measured and their wall thickness (W.Th) calculated [15]. On the trabecular bone surfaces, the percentage of quiescent surfaces (QS/BS), osteoid surfaces (OS/BS), eroded surface (ES/BS), and reversal surfaces (Rv.S/BS) per trabecular bone surfaces were estimated.

All histomorphometric parameters were compared with those of five healthy, age-matched women (aged 40–56 years) collected from a blinded biobank at Department of Forensic Medicine, Aarhus University.

### Combined Chromogenic in situ Hybridization and Immunostaining

The 3.5-µm-thick paraffin sections from the cortical bone of three coxa valga patients—which are considered to have a normal intracortical bone remodeling—were in situ hybridized with selective probe pairs for *FLNB* (832,691, ACD bioscience, Hayward, CA, USA) and detected using the RNAScope 2.5 high-definition procedure (310,035, ACD bioscience), enhanced by tyramide signal amplification (TSA) and chromogenic visualized using Liquid Permanent Red (Agilent, Santa Clara, CA, USA), as previously described in detail [16]. The sections were then immunostained for the osteoclast marker tartrate-resistant acid phosphatase (TRAcP) using mouse TRAcP antibodies (clone 9C5, MABF96, Merck Millipore, Hellerup, Denmark), detected with peroxidase-conjugated anti-mouse IgG polymers (BrightVision, Immunologic, Duiven, Holland), and visualized with Deep Space Black (Biocare Medical, Concord, CA, USA). Finally, the sections were counterstained with Mayer’s hematoxylin and scanned using the NanoZoomer XR digital slide scanner for imaging.

## Results

### Study Subjects

#### Subject 1 (Mother)

**Fig. 1:**
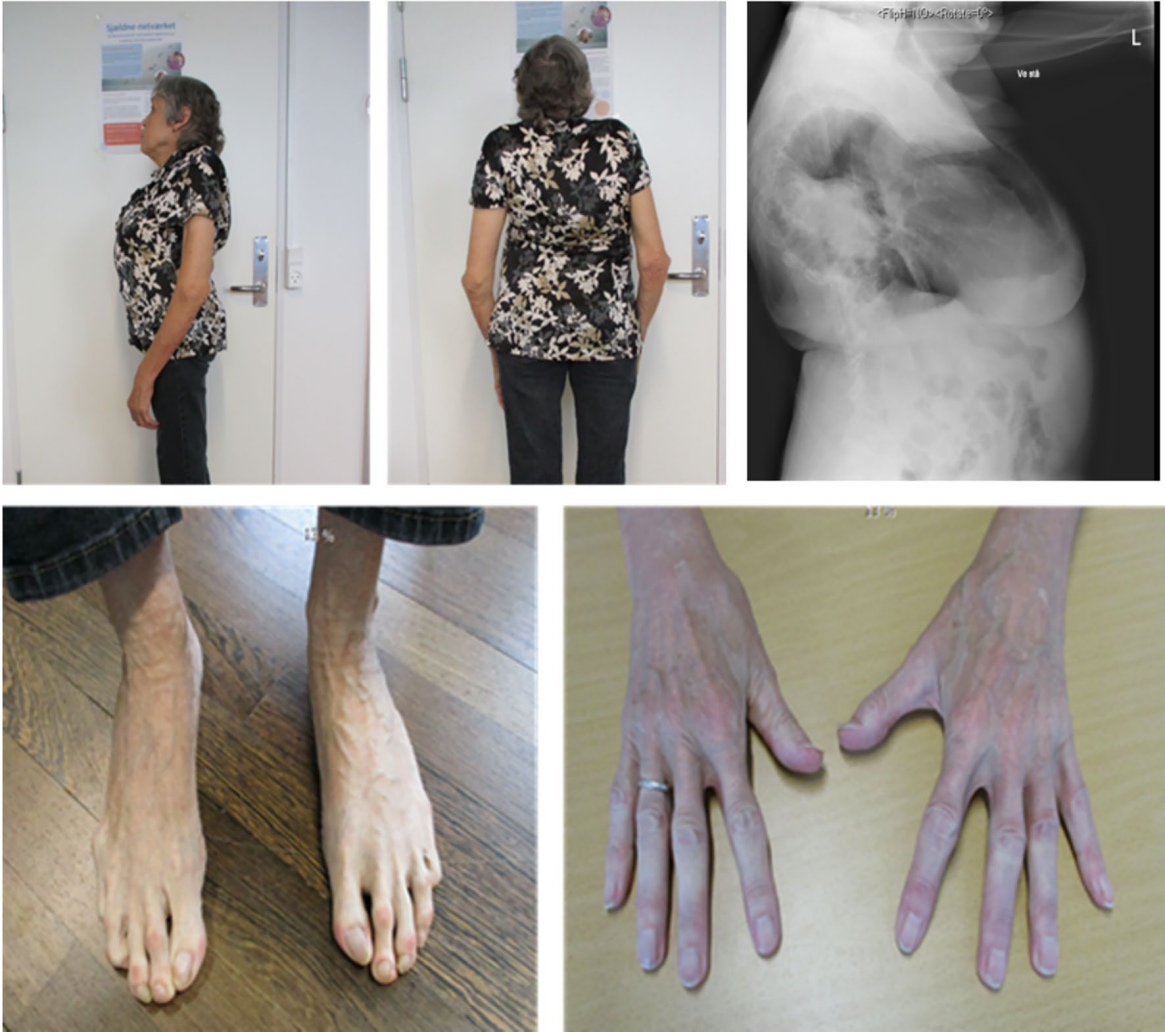
Subject 1. (**A**–**B**) The patient was clinically known to have kyphoscoliosis, pes planus, pectus carinatum, spatulate thumb, and hypermobility. (**C**) Radiographic lateral projection showing a chest X-ray). (**D**–**E**) Both hands and feet appeared elongated

The index case (subject 1), a 63-year-old with a height of 150 cm, developed progressive and severe kyphoscoliosis starting in her early teenager years. Additionally, she exhibited pes planus, pectus carinatum, spatulate thumb, and joint hypermobility (Fig. 1).

**Fig. 2:**
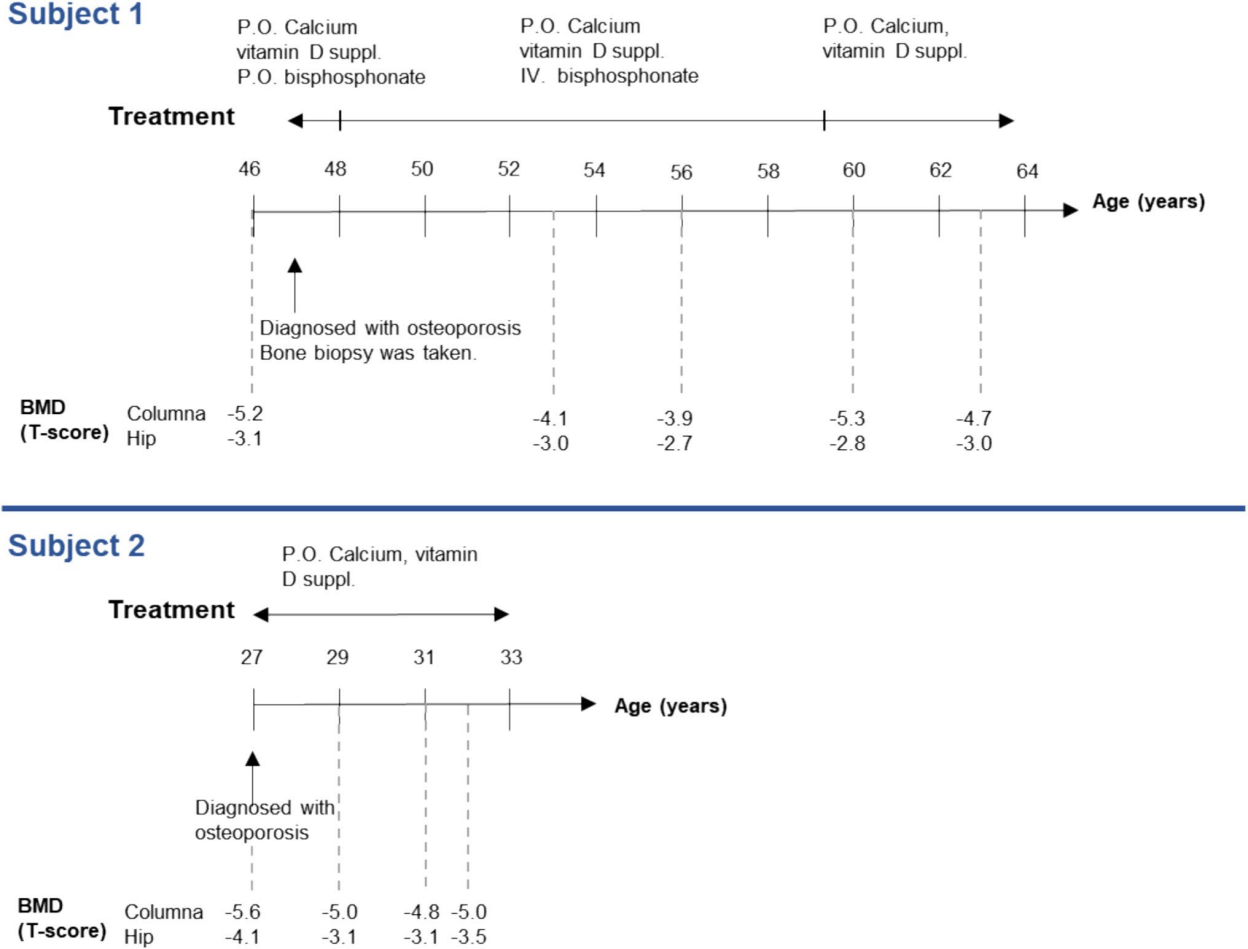
Timeline for Subject 1 and Subject 2, including age at diagnosis, medical investigations, medical treatment, and bone mineral density (BMD) measurements over time. Oral bisphosphonate refers to alendronate and IV bisphosphonate refer to zoledronate

In addition, she had a bilateral conductive hearing loss and began using hearing aids in her early forties. Her medical history included multiple hospital admissions due to reduced lung function (estimated as 40% of the expected value) and recurrent episodes of pneumonias. At the age of 46, a DXA scan revealed a spinal T-score of − 5.2 and a hip T-score of − 3.1 (Fig. 2). The subject experienced two high-energy fractures of the elbow due to falls but had no history of low-energy fractures. Risk factors included early menopause at the age of 35 and use of both oral and inhalation corticosteroids for lung infections and asthmatic symptoms. The subject was initially treated with oral bisphosphonates (alendronate, 70 mg per week) along with calcium and Vitamin D supplements for about one year. However, due to side-effects, the treatment was changed to intravenous bisphosphonate (zoledronate). Subject 1 underwent bisphosphonate treatment for a total of 13 years. While there was a minor increase in spinal BMD, no effect was observed in the hip BMD (Fig. 2). Biochemical assessments conducted at ages 46–47 (around the time of the bone biopsy) and 63 years of age were all within normal ranges (Table 1).

**Table 1:** Biochemical status of subject 1, taken at age 46–47 years (around the time of the bone biopsy) and age 63 years and subject 2 at age 33 years

	Subject 1 MotherAge 46/47 years	Subject 1MotherAge 63 years	Subject 2 DaughterAge 33 years
Calcium, ionized (mmol/L)	1.25 (1.19–1.29)	1.25 (1.18–1.32)	1.24 (1.18–1.32)
Phosphorus (mmol/L)	1.05 (0.78–1.58)	1.34 (0.76–1.41)	−
Alkaline phosphatase (U/L)	136 (80–275)	48 (35–105)	71 (35–105)
25-OH vitamin D (nmol/L)	58 (10–80)	133 (50–160)	104 (50–160)
Parathyroid hormone (PTH) (ρmol/L)	1.4 (1.4–6.9)	3.1 (1.1–6.9)	3.1 (1.1–6.9)
Carboxy-terminal collagen crosslinks (CTx) (µg/L)	−	0.17 (< 0.83)	−
Procollagen I intact N-terminal Propeptide (P1NP) (µg/L)	−	33 (13–116)	−

#### Subject 2 (Daughter)

The daughter (subject 2), a 33-year-old woman with a height of 158 cm, had congenital deafness in the right ear and a congenital right-side hip dislocation. She exhibited facial dysmorphology, including prominent supraorbital ridges, downward-arching upper eyelid, and retrognathia. Additionally, she had long hands and feet as well as a kyphoscoliosis. The subject also presented with visual impairment in the right eye, characterized by a reduced field of view and astigmatism.

Prior to the current genetic evaluation, she was diagnosed with Gorlin-Cohen syndrome (frontometaphyseal dysplasia) (OMIM 305620 and 617137) at the age of 15 [17], a condition that can clinically mimic Larsen syndrome.

At the age of 27, a DXA scan revealed severe low BMD, with a T-score of − 5.6 in the lumbar spine and − 4.1 in the hip (Fig. 2). The subject had a history of a forearm fracture from a bike accident but no prior low-energy fractures. Biochemically, she had severe Vitamin D deficiency and secondary hyperparathyroidism. She reported a very restricted diet consisting mainly of white bread and cereals. Her body mass index (BMI) was 23.5 kg/m^2^, and she had a regular menstrual cycle while using birth control pills. After treatment with calcium and Vitamin D, along with dietary changes, her levels of 25–OH Vitamin D, parathyroid hormone, and alkaline phosphatase all normalized (Table 1). However, her BMD remained low, with a lumbar spine T-score of -5.0 and a hip T-score of − 3.1 at age 29 (Fig. 2). Treatment with alendronate and intravenous bisphosphonate was considered, but at the patient’s request, these treatments were not initiated.

### Additional Family History

**Fig. 3:**
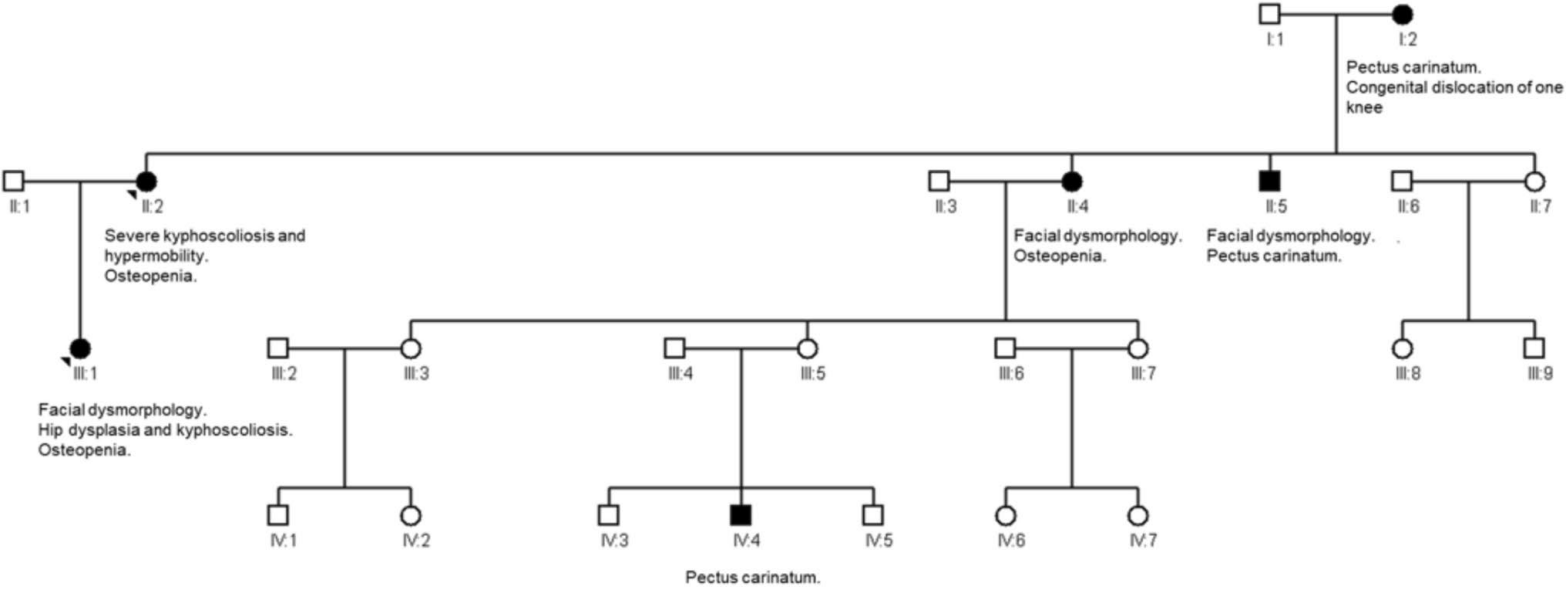
Family Tree of Subject 1 and subject 2. Clinically affected family members are marked with black. Subject 1: II:2, Subject 2: III:1. Squares represent males, and circles represent females

The brother (II:5) of subject 1 was reported to have pectus carinatum and facial dysmorphology. A sister (II:4) was described as having similar facial dysmorphology and osteoporosis, while a grandchild (IV:4) was born with pectus carinatum. The mother (I:2) had pectus carinatum and she was hospitalized for several months during her childhood due to congenital dislocation of one knee needing immobilization and plaster cast treatment (Fig. 3).

### Genetic Screening

Subject 2, initially clinically diagnosed with Gorlin-Cohen syndrome, was referred for genetic verification. However, a genetic screening of genes associated with Gorlin-Cohen syndrome (*MAP3K7, TAB2* and *FLNA*) was normal. Subsequently, whole exome sequencing was performed on both subject 1 and subject 2. This analysis identified a heterozygous missense variant in the *FLNB* gene; NM_001457.3: c.688G > T, p.(Val230Phe) in both subjects.

The c.688G > T variant is located in a highly conserved domain and has not been previously reported in gnomAd (Genome Aggregation database, https://gnomad.broadinstitute.org/). Other missense variants known to cause Larsen syndrome have been described within the same domain. Given the positive family history and the clinical phenotypes, the variant was classified as likely pathogenic (C4), and both subjects were diagnosed with Larsen syndrome.

### Evaluation of Bone Microarchitecture and Remodeling

The evaluation of the skeletal phenotype from a microarchitecture and remodeling perspective included: i) HR-pQCT of the distal tibia and radius (Subject 1 at age 63 years and subject 2 at age 33 years), ii) µCT and bone histomorphometry of a bone biopsy from subject 1, taken at the time of her osteoporosis diagnosis at age 47 years prior to bisphosphonate treatment, and iii) biochemical serum bone turnover markers (subject 1 at age 63 years). In addition, the physiological in situ expression of *FLNB* were investigated in human femoral bone samples from adolescent controls.

### Microarchitecture of Tibia and Radius Assessed by HR-pQCT

**Fig. 4:**
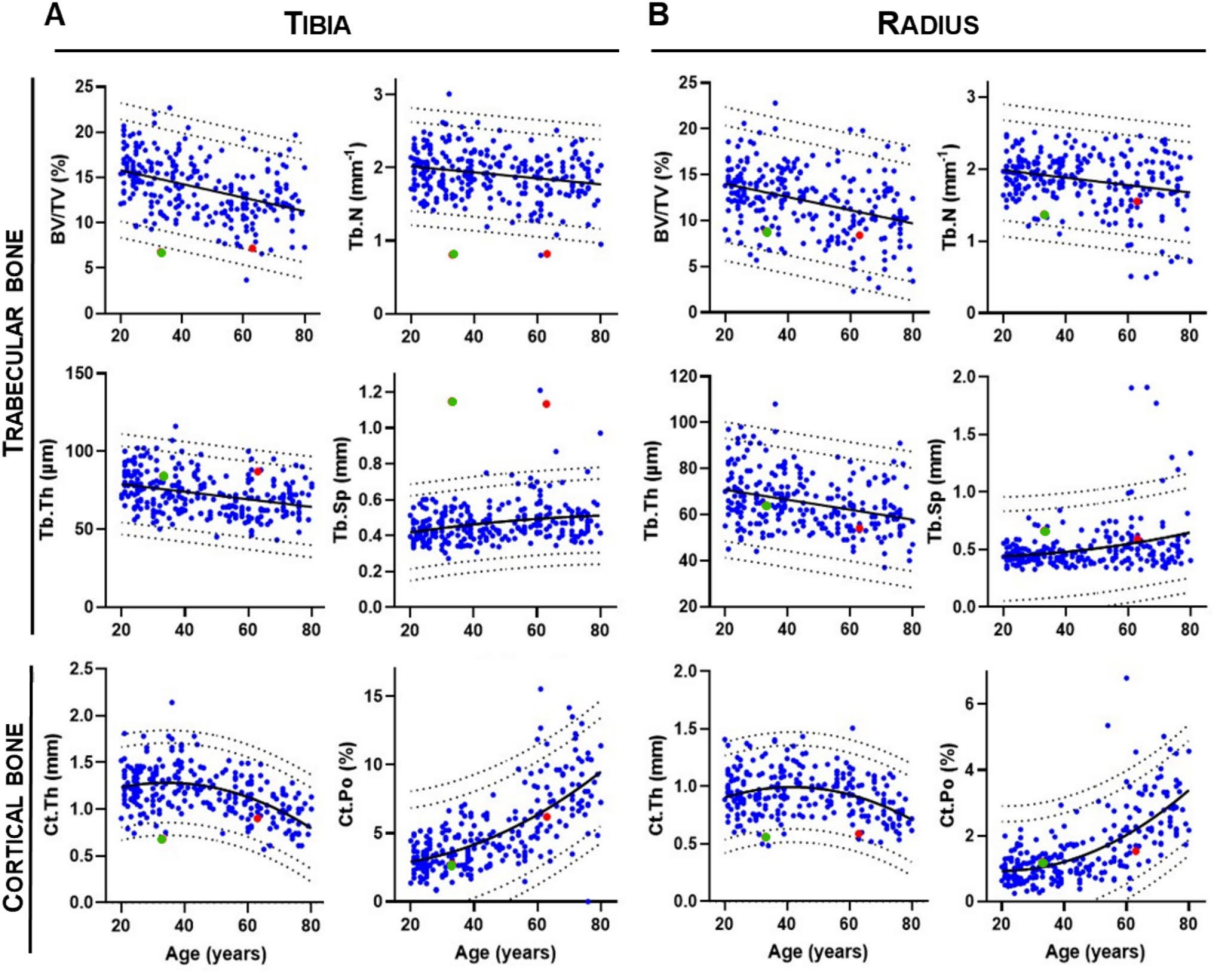
Microstructural parameters of the distal tibia (**A**) and radius (**B**) obtained by HR-pQCT for subject 1 (age 63) (red dots) and subject 2 (age 33) (green dots) compared with that of a healthy reference population of Danish women (blue dots) [9]. The solid line represents the best-fit curve for the reference population, and the dashed lines represent the 95 and 99% prediction bands

HR-pQCT analysis was used to investigate the bone microarchitecture of both the distal tibia and radius, compared to data from a reference group of Danish women [9] (Fig. 4). In the distal tibia, both subjects exhibited low trabecular bone volume (BV/TV), with subject 2‘s value falling outside the 99% predictive band. This appeared to be related to a reduced trabecular number (Tb.N) and increased trabecular separation (Tb.Sp), both of which were outside the 99% predictive band in both subjects (Fig. 4A). Cortical thickness was low in subject 2, but not subject 1, while the cortical porosity was normal (Fig. 4A). In distal radius, both subjects had normal trabecular and cortical parameters, except for the cortical thickness in subject 2, which was lower than the 95% predictive band, similar to the finding in the tibia (Fig. 4B).

### Microarchitecture of Bone Biopsy Assessed by µCT

**Fig. 5:**
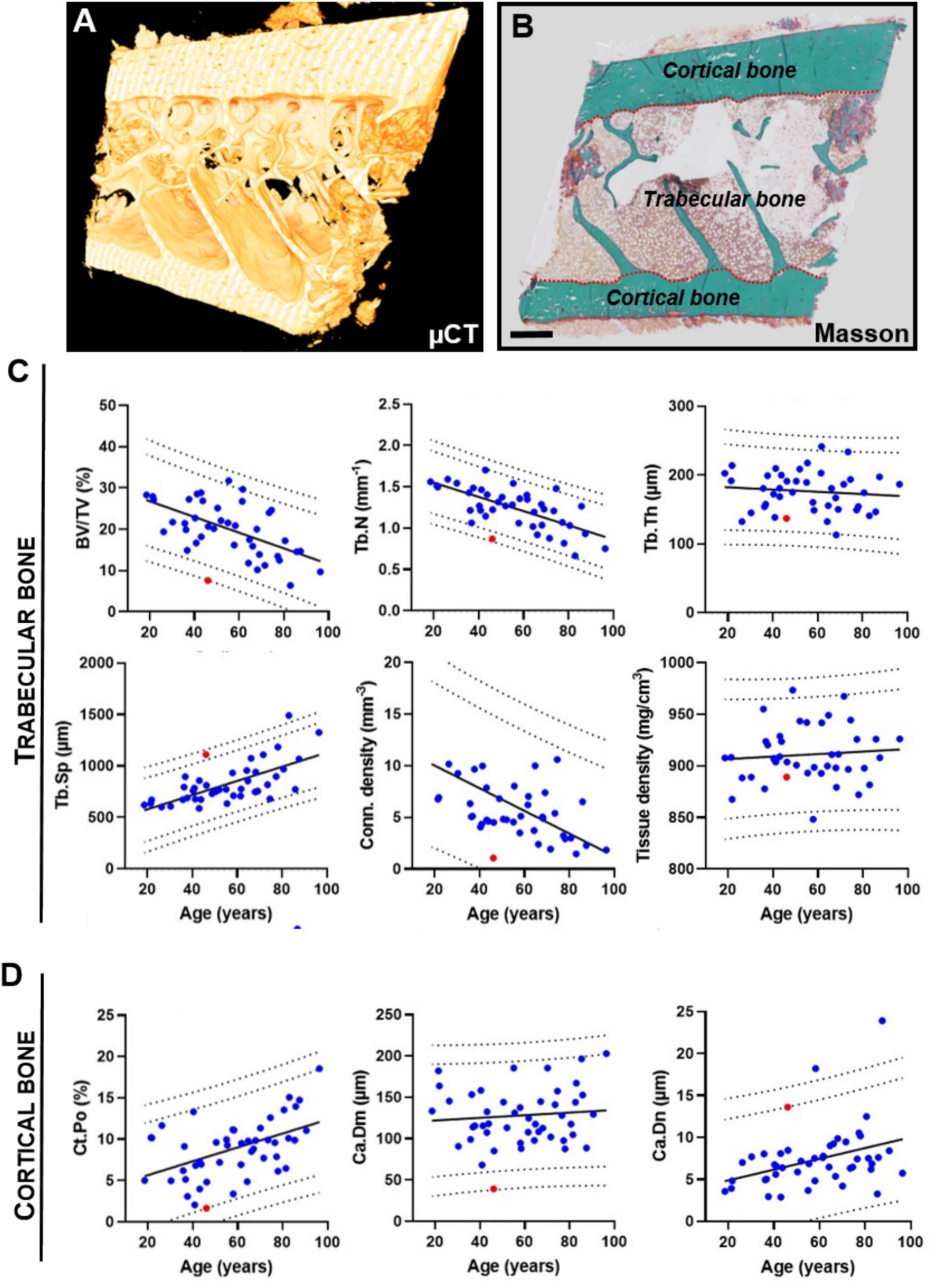
**A** µCT image and corresponding (**B**) Masson–Goldner trichrome-stained histological section of the bone biopsy from subject 1, with the border between the cortical and trabecular marked by a red dotted line. (**C**) Trabecular and (**D**) cortical 3D microstructural parameters for subject 1 (red dot) compared with those of a reference population of healthy Danish women (blue dots) [9]. The evaluated 3D microstructural parameters included trabecular bone volume fraction (BV/TV), trabecular number (Tb.N), trabecular thickness (Tb.Th), connectivity density, tissue mineral density, cortical porosity (Ct.Po), mean canal diameter (Ca.Dm), and canal density (Ca.Dn). The line represents the best-fit curves for the reference population, and the dashed lines represent the 95% and 99% prediction bands

A transiliac crest bone biopsy was obtained from subject 1 at age 47 years (around the time she was diagnosed with osteoporosis, but prior to bisphosphonate treatment). The original pathology report described the biopsy as having low trabecular bone volume, which was interpreted as severe osteopenia. In the present study, the remains of the embedded bone biopsy underwent µCT analysis to investigate its 3D microarchitecture, with comparison made to a reference group of Danish women [12, 13] (Fig. 5). In the trabecular bone compartment, subject 1 exhibited a low trabecular bone volume (BV/TV), which appeared to result from a low trabecular number (Tb.N) and increased trabecular separation (Tb.Sp) compared to the 95% prediction band of the reference population (Fig. 5C). Trabecular parameters such as trabecular thickness (Tb.Th), connectivity density, and tissue mineral density were not different from the reference population (Fig. 5C). In the cortical compartment, subject 1 exhibited low cortical porosity (Co.Po) and mean canal diameter (Ca.Dm), as well as high canal density (Ca.Dn), compared to the 95% prediction band of the reference population (Fig. 5D).

### Bone Remodeling Assessed by Histomorphometry and Biochemical Bone Markers

**Fig. 6:**
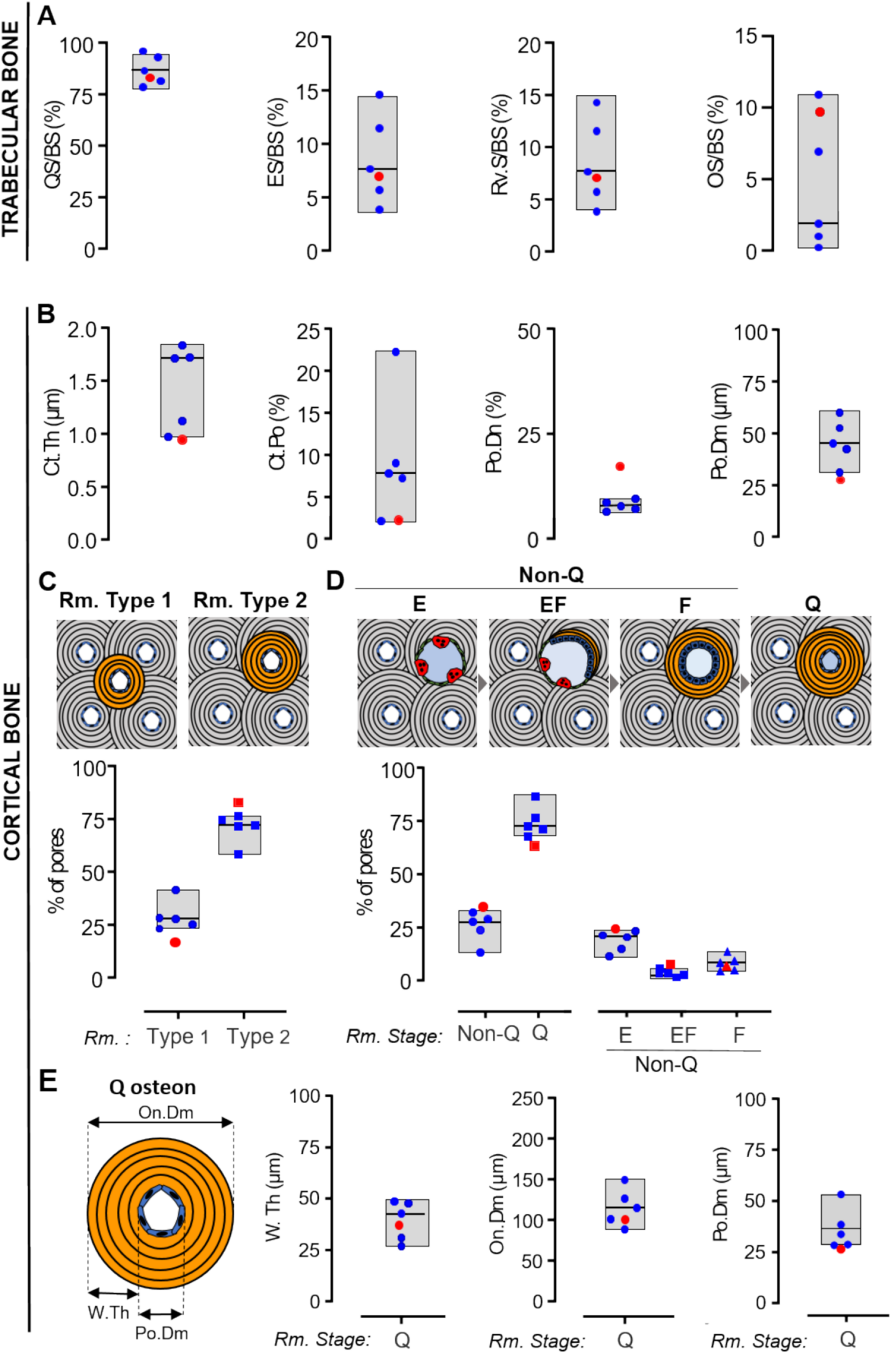
Trabecular (**A**) and cortical (**B**–**E**) bone histomorphometry of the transiliac bone biopsy obtained from subject 1 (red symbols) compared to age-matched female controls (blue symbols). (**A**) The trabecular bone surfaces were evaluated for the prevalence of quiescent surfaces (QS/BS), eroded surface (ES/BS), reversal surfaces (Rv.S/BS), and osteoid surfaces (OS/BS). (**B**–**E**) In the cortical bone, (**B**) the evaluated 2D microstructural parameters included cortical thickness (Ct.Th), cortical porosity (Ct.Po), pore density (Po.Dn), and pore diameter (Po.Dm). The intracortical pores were characterized according to their (**C**) remodeling type (Type 1—generation of new pores; Type 2—remodeling of existing pores), and (**D**) remodeling stage (Q—quiescent and Non-Q—non-quiescent, divided into E—eroded; EF—eroded-formative; F—formative pores). (**E**) The quiescent pores/osteons wall thickness (W.Th), osteon diameter (O.Dm), and pore diameter (Po.Dm) were evaluated. The gray box plot reflect the 95% prediction interval based on the reference

Masson–Goldner stained histological sections were used for detailed bone histomorphometry to assess remodeling activities within the trabecular and cortical bone compartments. The cortical bone was also analyzed for its 2D microarchitecture (Fig. 6). The original pathology report minimal to low bone remodeling activity and an increased number of intracortical Haversian canals. In the present study, trabecular histomorphometric analysis revealed that the percentage of quiescent (QS/BS), eroded (ES/BS), reversal (Rv.S/BS), and osteoid (OS/BS) surfaces in subject 1 was similar to those of the reference group of five healthy, age-matched women (Fig. 6A). The cortical histomorphometry of the 2D cortical microarchitecture revealed low cortical thickness (CT.Th), cortical porosity (Co.Po), and mean pore diameter (Po.Dm), as well as high pore density (Po.Dn) compared to the reference group (Fig. 6B). The cortical histomorphometry of bone remodeling showed i) less type 1 and more type 2 remodeling, ii) more non-quiescent and fewer quiescent pores, and iii) more eroded and eroded-formative pores compared to the reference group (Fig. 6C–D). Moreover, the quiescent pores/osteons showed no changes in the mean wall thickness (W.Th) and mean osteon diameter (On.Dm), but had a lower mean pore diameter of quiescent pores (Po.Dm) compared to the reference group. The lack of changes in trabecular bone remodeling and the minor changes in cortical bone remodeling align with both serum bone turnover markers CTx (resorption) and P1NP (formation), both of which were found to be within the reference range (Table 1). Note that the bone biopsy was taken at age 47 years, while the bone markers were measured at age 63 years, so they cannot be directly compared.

### In Situ Expression of *FLNB* in Human Bone

**Fig. 7:**
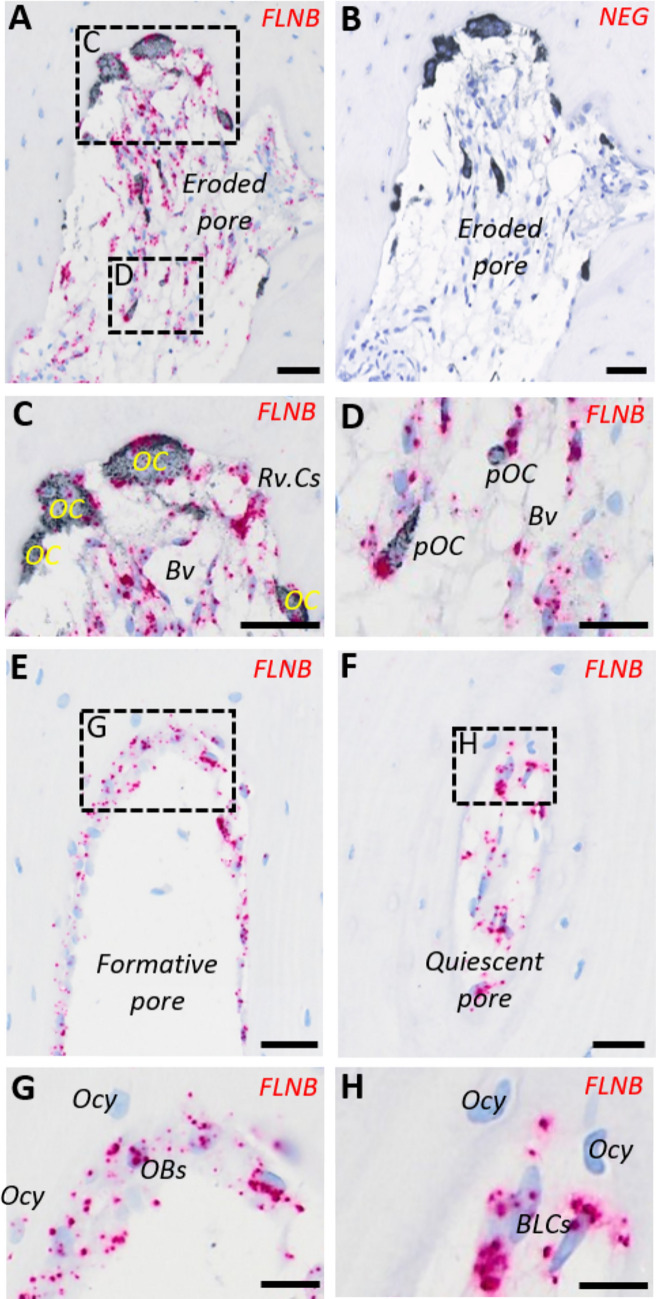
In situ hybridization (ISH) of filamin B (*FLNB*) mRNA expression (red dots) combined with immunostaining for the osteoclast marker TRAcP (black staining) in femoral bone specimens from a human adolescent control. (**A**–**B**) Images of adjacent sections of an eroded intracortical pore with ISH for (**A**) *FLNB* and (**B**) probe diluent as negative control. (**C**) High magnification images of bone-resorbing osteoclasts (OC), reversal cells (Rv.Cs), as well as (**D**) pre-osteoclasts (pOC), endothelial cells of blood vessels (BV) and neighboring osteoprogenitors in the pore lumen. Images of (**E**, **G**) formative pores and (**F**, **H**) quiescent pores, where high magnification shows in situ *FLNB* expression in (**G**) bone-forming osteoblasts (OB) and (H) bone-lining cells (BLCs), but limited expression in osteocytes (Ocy). Scale bars: 20 µm

In situ hybridization of *FLNB* combined with TRAcP immunostaining in femoral bone specimens from adolescent control subjects revealed broad expression of *FLNB* in all cells within the bone remodeling unit. This, included TRAcP^+^ osteoclasts, TRAcP^+^ pre-osteoclasts, reversal cells, osteoblasts, endothelial cells, osteoprogenitors, and bone-lining cells, with limited expression in osteocytes (Fig. 7).

## Discussion

Here, we report a new *FLNB* variant in two women with Larsen syndrome and provide a detailed description of their skeletal phenotype, which is characterized by low spinal and hip BMD, as well as reduced trabecular bone volume, trabecular number, and cortical thickness.

A 24-repeat encoding-region of *FLNB* is essential for the formation of Filamin B dimers [2], and the majority of heterozygotic pathogenic variants associated with Larsen syndrome cluster in this repeat region or in the actin-binding domain [2, 18]. The *FLNB* variant (c.688G > T p.(Val230Phe)), which replaces valine with phenylalanine, is predicted to induce a conformational change in the protein, affecting the actin-binding domain and resulting in a pathological impact on Filamin B function.

Both subjects, mother and daughter, exhibited remarkably low BMD at a young age, leading to their diagnosis of osteoporosis at the ages of 46 and 27 years, respectively. Although, BMD was low in both patients, neither had sustained fractures, and there was no family history of fractures. Importantly, both had known risk factors for low BMD, including corticosteroid treatment and early menopause (Subject 1), as well as nutrient-poor diet (Subject 2), which could independently contribute to reduced BMD [19, 20]. Of note, subject 1 was receiving hormone replacement therapy until the age of normal menopause. Therefore, we cannot exclude the possibility that part of their skeletal phenotype is secondary. Nevertheless, the observation of very low BMD across time in both subjects suggests that patients with Larsen syndrome are at risk of severe osteoporosis.

From a microstructural perspective, the reduced BMD in both subjects appears to result from low trabecular bone volume, driven by a decreased trabecular number, increased trabecular separation, and reduced cortical thickness—at least in the distal tibia and iliac crest. These microstructural characteristics are likely the result of altered skeletal development and growth, as *FLNB* knock-out mice exhibit delayed bone formation in the long bones, along with dwarfism and early fusion of the vertebral, carpal, and tarsal bones [21]. In humans, loss of function of *FLNB* leads to a similar skeletal phenotype with spondylocarpotarsal synostoses syndrome (OMIM 272460), while missense variants, such as the one investigated in the current study, lead to more heterogeneous phenotypes. These can range from severe forms with Boomerang dysplasia or Larsen syndrome to milder manifestations such as isolated scoliosis [3, 22, 23].

Larsen syndrome exhibits heterogeneous phenotypic presentations, even within the same family [1, 2, 24], which is in line with the findings from the present family. Upon more detailed analysis, both subjects present a very similar skeletal phenotype. In subject 2 (age 33), this phenotype could be interpreted as premature skeletal aging, significantly different from the reference population, while in subject 1 (age 63), it is more similar to the aging reference population. These findings support the notion that the skeletal phenotype primarily develop during skeletal development and growth, with less influence from changes in the bone remodeling process during adulthood.

Bone remodeling occurs within microscopic basic multicellular units (BMUs), which continuously renew the bone matrix throughout life [25]. The bone remodeling process involve four sequential phase: i) An activation phase, ii) an initial resorption phase, where primary osteoclasts convert quiescent bone surfaces into eroded bone surfaces, iii) a reversal-resorption phase, during which secondary osteoclasts deepen the erosion while intermixed with osteoprogenitors (reversal cells) that gradually proliferate and differentiate into bone-forming osteoblasts, and iv) a bone formation phase, where osteoblasts refill the eroded cavity [10, 26, 27]. During aging and osteoporosis, the transition from eroded surfaces to bone formation is delayed, leading to an accumulation of eroded cavities and subsequent bone loss [14, 28, 29].

In subject 1, histomorphometry of the bone biopsy, taken at the age of 47, revealed trabecular bone remodeling comparable to the age-matched female reference group. However, the cortical bone exhibited slightly more pores that were classified as either eroded or eroded-formative compared to the reference group. This suggest that the transition from eroded pores to formative pores is slightly delayed compared to reference group of age-matched healthy women. Typically, this delayed transition leads to increased cortical porosity and mean pore diameter [14]. However, in subject 1, we observed the opposite trend, with decreased mean pore diameter and increased pore density, indicating a greater number of smaller eroded and eroded-formative pores.

The measurements of quiescent pores/osteons allow us to investigate the extent of erosion (osteon diameter) and formation (wall thickness), as well as the BMU balance between erosion and formation (pore diameter) [15]. Subject 1 showed no changes in the magnitude of erosion and formation, but exhibited a slightly improved BMU balance (lower pore diameter) compared to the reference group of age-matched healthy women.

Overall, these subtle effects of the *FLNB* variant on bone remodeling at the time of investigation align with the later investigated biochemical bone markers (CTx and P1NP), which were within normal ranges. This suggests that neither overall bone turnover nor bone formation or resorption were altered, as typically the case in classic osteoporosis, where these markers are considered valid for monitoring the disease and its response to treatment [30]. However, we cannot exclude that the bone remodeling remains suppressed by the bisphosphonate treatment ending around 3 years prior to the bone turnover markers [31].

The *FLNB* gene, which encodes Filamin B, is essential not only for the skeleton but also for various other tissues [32, 33]. In healthy human bone, we observed broad in situ expression of *FLNB* in osteoblast-lineage cells, osteoclasts, and endothelial cells, consistent with previous in vitro studies and immunostainings for Filamin B [21, 34, 35]. In contrast, *FLNB* expression was limited in osteocytes. Specifically, we found *FLNB* expression in osteoprogenitors (reversal cells and cortical lumen cells) that gradually differentiate into bone-forming osteoblasts, playing a critical role in securing the transition from bone resorption to bone formation [10, 26]. The *FLNB* expression in osteoclasts and osteoprogenitors supports the notion that the slight delay in the transition from resorption to formation in subject 1, compared to the investigated reference group, might reflect a subtle effect the *FLNB* variant on this mechanism, which warrant further investigation.

A key question remains regarding the treatment of patients with Larsen syndrome. Subject 1 was treated with both oral and intravenous bisphosphonates, but there was no noticeable effect on the BMD. This could be because subject 1 did not exhibit a high rate of bone resorption, which is typically targeted by bisphosphonates. An alternative approach might involve bone-anabolic treatments, such as intermittent teriparatide (PTH 1–34) or romosozumab (anti-sclerostin antibody) [36]. One could speculate, if these treatment options may be more effective, as patients with Larsen syndrome often experience severe osteoporosis.

In conclusion, our findings support the view that Filamin B function is essential for normal bone morphology. The newly identified *FLNB* variant is associated with low BMD, reduced trabecular number, and decreased cortical thickness, further emphasizing the importance of FLNB in bone health.
